# A Multimodal Approach to Stratification of Patients with Dementia: Selection of Mixed Dementia Patients Prior to Autopsy

**DOI:** 10.3390/brainsci9080187

**Published:** 2019-08-01

**Authors:** Gary A. Rosenberg, Jillian Prestopnik, Janice Knoefel, John C. Adair, Jeffrey Thompson, Rajikha Raja, Arvind Caprihan

**Affiliations:** Department of Neurology, University of New Mexico Health Sciences Center and the MIND Research Network, Albuquerque, NM 87131, USA

**Keywords:** vascular cognitive impairment, Alzheimer’s disease, mixed dementia, biomarkers, cerebrospinal fluid, diffusion tensor imaging, neurodegeneration, neuroinflammation

## Abstract

Alzheimer’s disease (AD) and vascular cognitive impairment and dementia (VCID) are major causes of dementia, and when combined lead to accelerated cognitive loss. We hypothesized that biomarkers of neurodegeneration and neuroinflammation could be used to stratify patients into diagnostic groups. Diagnosis of AD can be made biologically with detection of amyloid and tau proteins in the cerebrospinal fluid (CSF) and vascular disease can be identified with diffusion tensor imaging (DTI). We recruited patients with cognitive complaints and made an initial clinical diagnosis. After one year of follow-up we made a biological diagnosis based on the use of biomarkers obtained from DTI, CSF AD, and inflammatory proteins, and neuropsychological testing. Patients with AD had primarily findings of neurodegeneration (CSF showing increased tau and reduced amyloid), while patients with neuroinflammation had abnormal DTI mean diffusion (MD) in the white matter. Using the biological biomarkers resulted in many of the clinically diagnosed AD patients moving into mixed dementia (MX). Biomarkers of inflammation tended to be higher in the MX than in either the AD or VCID, suggesting dual pathology leads to increased inflammation, which could explain accelerated cognitive decline in that group.

## 1. Introduction

The incidence of dementia is increasing, creating a worldwide health care crisis. Dementias are a heterogeneous group of brain disorders with overlapping clinical presentations [1,2]. To improve classification and to form more homogeneous groups of patients, many investigators are developing biomarkers for both classification and treatment trials. Without adequate ways of classifying this highly heterogeneous group of patients, development and testing of novel treatments will continue to be impeded. A number of biomarkers are under development that can be used to improve diagnosis [3,4]. These biomarkers are derived from multiple sources, including neuropsychological testing, magnetic resonance imaging (MRI) studies, and biochemical analysis of blood and cerebrospinal fluid (CSF). Although there are excellent biomarkers available, currently no single biomarker is adequate for separation of the various dementia types, making a multimodal approach necessary.

A number of recent large autopsy series of patients with dementia and healthy elderly have been published [5,6,7,8]. Most document a high percentage of patients with multiple pathological findings, with an estimated 70% of patients having both Alzheimer’s disease (AD) proteins and cerebrovascular disease (CVD). The first indication that a combination of pathological changes may be more important than one of them alone in causing cognitive loss came from the “Nun Study,” which showed that those with pathological evidence of both AD and CVD were most likely to have dementia [9].

Prior reports, by us and others, have detailed methods using multimodal biomarkers in the diagnosis of vascular cognitive impairment and dementia (VCID) [4,10,11]. With the development of diffusion tensor imaging (DTI), an MRI method that detects subtle microstructural white matter damage, it is possible to detect CVD as shown by microstructural damage to white matter, and with improved diagnosis of AD with biomarkers, it is now possible to identify patients with both AD and VCID prior to autopsy.

Pathological changes associated with AD can be ideally identified using the biological markers beta-amyloid (Aβ) and phosphorylated tau (Ptau) in CSF or positron emission tomography (PET), using radionuclide-labelled ligands that bind to these proteins [4,12]. Biomarkers derived from MRI provide important structural and physiological data while CSF or PET characterize underlying pathophysiologic process [13].

While both CVD and AD pathologies are commonly present, they generally follow a different time course. Deposition of Aβ begins up to several decades prior to the onset of cognitive symptoms [14]. Similarly, the pathological changes associated with CVD begin early in patients with hypertension [15]. While any individual biomarker is insufficient to distinguish different types of dementia, combining biomarkers improves classification [10,11]. The challenge of a classification method using biomarkers is to both identify patients with primarily AD or VCID as well as recognize patients with dual pathologies forming the subgroup of mixed dementia (MX). We hypothesize that biomarker combinations can separate patients, allowing identification of patients with MX during life. In this report, we describe a method to more precisely stratify patients into the major dementia pathophysiologic subtypes using a combination of biomarkers. We show that biomarkers for both VCID and AD can identify the group of patients with mixed pathologies that is the common form identified in autopsy series. We compare the standard clinical diagnoses with the biological diagnoses. This report describes the rationale for selecting biomarkers to identify MX patients in vivo.

## 2. Methods

### 2.1. Patients and Biomarkers

Patients in this report were recruited from the University of New Mexico Neurology Clinic and the Albuquerque Veterans Administration Hospital from 2006 to 2018 as described in detail in earlier reports [10,11]. They underwent neurological examinations and a battery of neuropsychological tests. After initial clinical evaluation, investigators rendered consensus clinical diagnoses based on all clinical and neuropsychological data and review of their routine brain MRI. Clinical diagnoses were: (1) subcortical ischemic vascular disease (SIVD), the small vessel form of VCID that is called by some investigators, Binswanger’s disease (BD) [16,17,18]; (2) patients with multiple infarcts (MI) due to large vessel or single strategic strokes; (3) Alzheimer’s disease (AD) was diagnosed using criteria described in earlier reports [19,20]; (4) when both vascular disease and AD were thought to be present, MX was used; and (5) a fifth group included patients with white matter changes on fluid attenuated inversion recovery (FLAIR) MRI that were not necessarily pathological (i.e., normal neurological and neuropsychological status). This last group was called leukoaraiosis (LA) as originally defined to represent “rarefied white matter” on neuroimaging [21]. The classification was based on published clinical criteria and the consensus of the three study neurologists [22].

### 2.2. Stratification Based on Biomarker-Based Information

A second classification was done with patients stratified according to biologically-based biomarkers obtained from the research MRI and biochemical analysis of the CSF [4]. Measurements of AD proteins, ratio β-amyloid_1–42_/β-amyloid_1–40_ (Aβ_1–42_/Aβ_1–40_) and Ptau, were utilized for the biomarker-based classification of AD. In addition, we used mean diffusivity (MD) from DTI of white matter to indicate structural damage as a biomarker of vascular injury.

### 2.3. Cognitive, CSF, and MRI Assessments

Cognitive tests were administered by a trained research psychologist (JP) and scored according to standard procedures. Standardized (T) scores were calculated for each test. Averaged composite T-scores were calculated for separate cognitive domains: memory (Hopkins Verbal Learning Test-Delay, Rey Complex Figure Test-Long Delay), executive function (Digit Span Backwards, Trail Making Test B, Stroop, Controlled Oral Word Association (FAS)), attention (Digit Span Forward and Trial Making Test A), language (Boston Naming 60 item test, Controlled Oral Word Association (Animal)) and processing speed (Digit Symbol and Symbol Search, both based on WAIS-III). An overall cognitive composite score was derived as the mean of individual domain T-scores. Control participants for the MRI studies underwent the same neuropsychological test battery.

CSF biomarkers were obtained by lumbar puncture performed by one of the authors. Samples were centrifuged, aliquoted, and stored at −80 °C for later analysis. Two enzyme-linked immunoabsorbant assay (ELISA) kits were used to measure Aβ_1–42_/Aβ_1–40 _(Meso Scale Discovery, Rockville, MD) and Ptau (Fujiribio Malvern, PA). Control CSF was obtained from patients undergoing spinal anesthesia for orthopedic surgery. Matrix metalloproteinases were measured by ELISA (Meso Scale Discovery).

MRI scans were performed on a Siemens 3T scanner with the initial scans performed on a 12-channel radio frequency (RF) coil and later scans were done with a 32-channel RF coil. The imaging parameters with the two RF were closely matched. The 3D MPRAGE sequence had TR = 2530 ms, four echoes, and TI = 1200 ms with an acquisition time of 6.5 min. The 3D FLAIR sequence had a TR = 6000 ms, TE = 427 ms, and TI = 2000 ms. The diffusion data was collected with a FOV = 2242 mm isotropic resolution, and 72 slices for both RF coils. On the 12-channel coil, the diffusion protocol had a single-shell of b-value = 800 s/mm^2^ with 30 volumes collected with different gradient directions and five volumes with b = 0. The acquisition time was 6.5 min. The experiments done on the 32-channel coil used a CMRR multi-band sequence, which enabled us to collect more gradient directions in an equivalent experiment time. On the 32-channel coil we collected three shells with a maximum b-value = 3000 s/mm^2^, 155 volumes with different gradient directions, and eight volumes with b = 0. The acquisition time was 12.5 min.

White matter hyperintensity (WMH) volume was calculated from FLAIR images based on JIM software (www.xinapse.com). The diffusion images were corrected for motion, distortion, and mean diffusivity (MD) and fractional anisotropy (FA) were calculated (www.fmrib.ox.ac.uk).

### 2.4. Statistical Analysis

The overall group difference was tested with a non-parametric Kruskal-Wallis ANOVA test. Mann–Whitney U-test was selected to test for pairwise group differences. The corresponding *p*-values were corrected for multiple comparisons.

## 3. Results

Clinical diagnoses were made initially on all patients, placing them in one of five groups. The clinical diagnoses were based on clinical information, including neuropsychological test results and routine MRI, primarily FLAIR. All of the information was obtained in the clinical visit. Differentiation of patients using clinical criteria alone provided information on the ability of the clinician using data available from an office visit.

After one-year follow-up, a biological diagnosis was made that included information from the biomarkers from the research MRI and CSF biochemical studies. The MRI data that was used came from MD from DTI. The biomarker features that were included in the statistical analysis were developed as part of a study of biomarkers in SIVD, AD and MX, and were part of a machine learning study of Binswanger’s disease [11]. A list of the features used in the analysis with their presence or absence is shown in Table 1.

Clinical features that were useful were increased reflexes, which could be asymmetric. This suggested white matter damage as seen on standard MR imaging in SIVD and MX groups. Neuropsychological testing could be divided into several major categories. Executive dysfunction was most important in the SIVD group and showed variable results in the other groups, but could not separate patients. Memory was a more reliable biomarker since it was consistently reduced in AD and MX. MRI biomarkers were important in showing injury to white matter. The WMHs seen on clinical MRI FLAIR sequences were nonspecific and observed in SIVD, MX, and LA groups. Diffusion biomarkers obtained from DTI measurements of MD were more reliable and several of the diagnostic groups changed when the biomarker-based classification system was used.

Classification of patients using clinical diagnoses and biological biomarker diagnoses is shown in Table 2. Using biomarkers resulted in a decrease in the number of AD patients and an increase in MX. This was mainly due to finding of abnormal DTI values that suggested a vascular contribution to clinically diagnosed AD patients. CSF measurements of Aβ_1–42_/Aβ_1–40_ ratio was reduced and Ptau elevated in the AD and MX groups. While none of the biomarkers could be used independently for classification, the combination of multiple biomarkers was effective.

**Figure 1:**
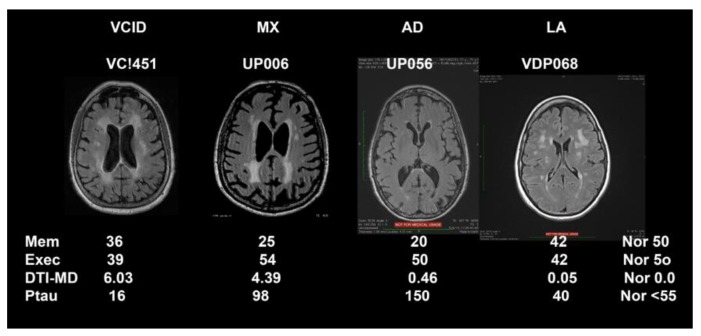
MRI fluid attenuated inversion recovery (FLAIR) sequences on top and biomarker values for each of the patients from the different diagnostic categories. The vascular cognitive impairment and dementia (VCID) patient has a normal memory and executive score. The diffusion tensor imaging-mean diffusion (DTI-MD) is elevated with a normal of 0.0 since these are z-scores, and the Ptau is normal. The mixed dementia (MX) patient shows a low memory and normal executive function, an elevated DTI-MD, and an increased Ptau. The Alzheimer’s disease (AD) patient has very low memory score with a normal executive function, a normal DTI-MD and an extremely elevated Ptau. The leukoaraiosis (LA) patient has white matter changes on FLAIR, but normal scores for all the biomarkers, including MD. Normal (Nor) values are shown in the right column.

Representative FLAIR MRIs along with biomarker results that were used to form the biological data are shown in Figure 1 for the four major classification categories (MI not shown). This figure also emphasizes the importance of DTI to show pathological changes in the white matter water since SIVD, MX, AD, and LA appeared similar on FLAIR MRI.

Using biomarkers for patient stratification into SIVD, MX, AD, and LA, differences between diagnostic categories becomes evident. This can be seen in data shown in Table 3. The MX patients tended to have higher values than other groups in most areas. The age of the patients with dual pathologies is higher than in other groups. T-scores for executive function are lowest in the SIVD group due to the damage to white matter, but executive function scores are not able to separate patients well. Memory function, on the other hand, is more clearly delineated with the lowest scores seen in the AD patients, and also in MX. The other neuropsychological tests failed to provide clear separation of the patients.

The diffusion results were very useful in the stratification. We formed z-scores for the MD values so that normal would be close to zero and elevated values would be in the 1 to 3 range. DTI MD was markedly elevated in SIVD and MX groups indicating severe damage to the white matter. More importantly, MD values in LA were similar to AD and slightly elevated compared to the normal range in spite of large WMHs on FLAIR images. This demonstrates that FLAIR MRI is an unreliable measure of white matter damage since it does not clearly delineate the structural state of the tissue.

The results of CSF biochemical studies were mainly important in the biological definition of AD and in showing those patients with dual pathologies as indicated by elevated MD. Ptau was increased in both MX and AD patients (Table 3). We found differences in the inflammatory state as measured by the MMPs in the CSF. MMP-1 was highest in the SIVD, suggesting that there was inflammation, although the results were not statistically significant. There was an elevation of MMP-3 in MX, but not in AD. The most striking change in the MMPs was observed in the significantly elevated levels of MMP-10 in MX and AD.

## 4. Discussion

Our results show that separation of patients with biomarkers identifies a group of MX patients during life. We found a large number of patients that could be classified as having dual pathologies, which is consistent with a recent large autopsy series from Alzheimer’s Disease Research Centers that have shown the high incidence of patients with dual pathologies, primarily related to AD protein deposition and CVD with or without infarcts [6,8]. While autopsy data is the “gold standard” diagnostic method, it represents the accumulation of a lifetime of pathology. The use of biological biomarkers for patient stratification has the benefit of revealing the pathological changes during life, which can inform treatment. We used a combination of biomarkers derived from DTI and CSF to diagnose patients with dual pathologies. Cerebrovascular disease was identified by abnormalities in MD on DTI, indicating underlying structural damage in the white matter, which were suggestive of neuroinflammation. The presence of AD proteins in the CSF indicated neurodegeneration. These showed the two major pathways to pathology leading to dementia. With the use of these biomarkers, patients with WMHs associated with ”normal” aging could be determined, leading to more homogeneous patient populations through the exclusion of such patients.

The AD diagnoses benefit from the use of CSF for biological diagnosis [4]. In our cohort of patients with cognitive impairment, we were able to improve diagnostic accuracy by increasing the number of patients with the diagnosis of MX and confirming the lack of white matter injury in LA. It is possible to discriminate patients with mainly vascular disease using DTI and degenerative disorder (specifically AD) by CSF. From the results of autopsy studies, we would anticipate that the number of AD and SIVD is smaller compared to those with joint pathology [6]. When the AD proteins are present and the DTI is normal, the patient had primary AD. Likewise in the patient with abnormal white matter DTI and normal AD proteins in the CSF, the primary diagnosis is SIVD.

We have made an assumption that the findings on DTI are indicative of vascular disease. While this is true, there are a large number of other disease processes that lead to white matter damage, making DTI a nonspecific marker. Interpretation of the results of DTI should include other information that place the patient in the vascular category. This is the reason that the combination of MRI and CSF data is critical.

**Figure 2:**
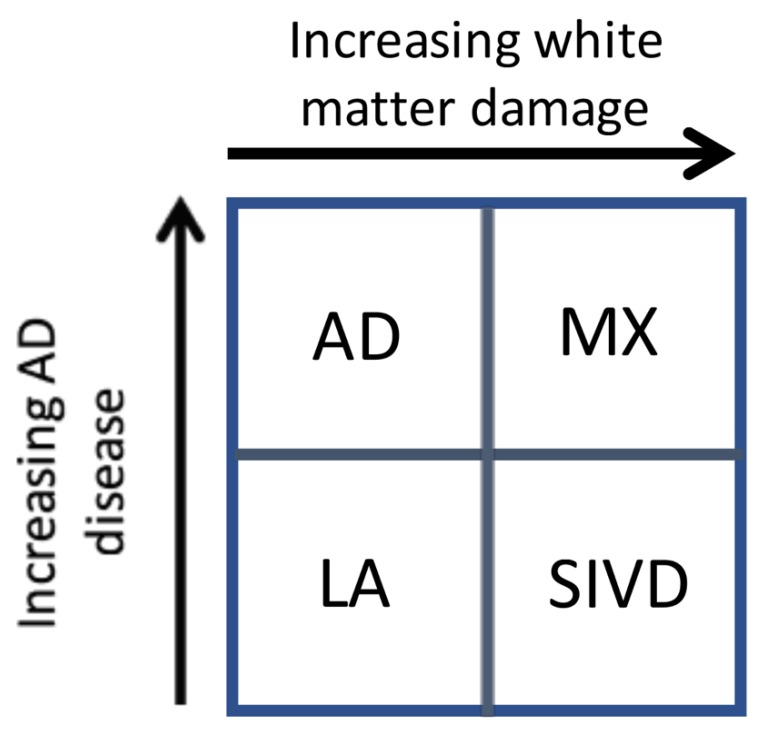
Classification of patients into diagnostic groups with a double dichotomy approach based on CSF studies and MRI DTI being on separate axes. On the *y*-axis there is increasing values for CSF Alzheimer protein with low levels of leukoaraiosis (LA) and high levels in Alzheimer’s disease (AD). On the *x*-axis, there are increasing values for MRI DTI with the lowest values in the LA group and the highest values in subcortical ischemic vascular disease (SIVD). When the DTI is high and the CSF proteins are high, the patient is diagnosed as mixed dementia (MX).

**Figure 3:**
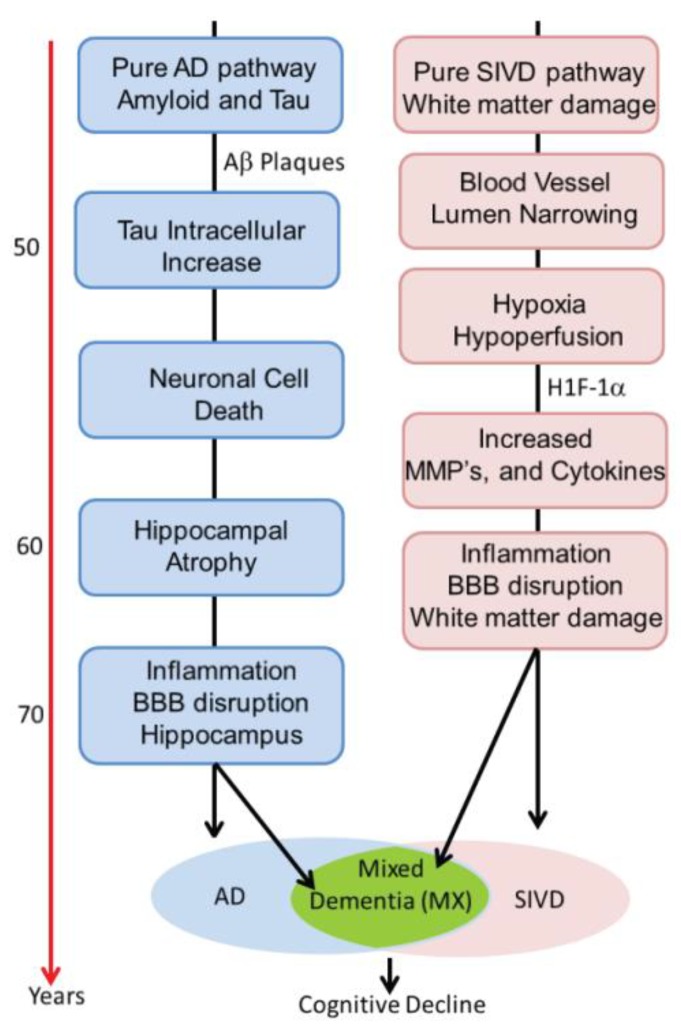
The concept of dual pathways leading to mixed dementia is shown schematically. On the left is the Alzheimer pathway. This begins early with deposition of amyloid and proceeds through stages of phosphorylated Tau, cell death, and atrophy. On the right the vascular pathway is shown, which also begins early with vascular risk factors that are poorly controlled. When the two pathways converge later in life, they form mixed dementia (MX). At this stage, there already is on-going inflammation from each pathway alone, which now is exaggerated. HIF-1α is hypoxia inducible factor-1α.

Our results suggest that there are two major dichotomies that lend themselves to separate analysis. This is shown in the schematic diagram in Figure 2. The first is shown on the *y*-axis, where increasing evidence of the diagnosis of AD is based on the results of CSF studies. Similarly, the *x*-axis shows the impact of white matter injury as shown by DTI, but not necessarily FLAIR MRI. As white matter becomes more severely injured, diagnostic certainty of SIVD increases. The importance of this approach is two-fold. First, it implies that the patients with dementia can follow one of two pathways. In the AD pathway, the deposition of amyloid, leading to increases in Ptau and eventually to cell death and brain atrophy constitutes one pathway. Similarly, the vascular pathway begins at a similar early age with damage to the blood vessels. This can be due to hypertension, diabetes, smoking, and other known vascular risk factors and this pathway slowly progresses in a parallel fashion. At some point, there can be the onset of inflammation from either the amyloid or dying cells with microglial activation or from the damaged blood vessels, which attempt to remodel though the use of proteolytic enzymes released by microglia or invading macrophages. The consequences of this dual pathology culminate by increased inflammation and the diagnosis of mixed pathology can be made [22]. This process is illustrated schematically in Figure 3. Alzheimer’s disease has inflammation thought to be related to activation of microglia around the amyloid plaques. Inflammation is also prominent in human brains with SIVD. We propose that there are two pathways active in the patients with dementia. One is the amyloid/tau mediated neurodegenerative pathway that can begin in middle age and progress slowly over decades. The other is the vascular pathway initiated by blood vessels damaged by long-standing poorly controlled hypertension and diabetes. Both pathways can progress independently for many years. It is also possible that the two pathways interact at earlier stages (e.g., via amyloid angiopathy). With advancing age, however, the pathways may merge, creating a more severe form of inflammation. This is suggested by data from the ”Nun study”, where the presence of both pathological processes accelerate cognitive decline. We have used the schematic approach to describe a complex process that involves other causes of dementia. Also, the point at which the two processes converge is highly variable.

Matrix metalloproteinases are a large group of proteases active against the extracellular matrix [23,24]. We have demonstrated that MMPs are associated with inflammatory reactions that lead to disruption of the blood-brain barrier [25]. We found that MMP-1 was highest in SIVD suggesting a role in inflammation, while MMP-3 and MMP-10 were highest in AD. Others have observed elevated MMP-10 in AD, but the significance of this observation is unclear [26]. By separating patients into more homogeneous groups, the increase in white matter MD can be seen in the SIVD and MX groups, but not in AD and LA. As expected, Ptau was high in AD and MX.

A caveate in the use of clinical and biomarker diagnoses is lack of a “gold standard”. Our purpose in this study was to determine the ability of the clinician to separate patients on the basis of the limited information available in the clinic. Comparing this to the biological biomarker approach revealed the benefit of this additional information. We recognize the circularity involved in this approach. However, now that we have identified the appropriate biomarkers for a better patient stratification, these can be used in research studies.

## 5. Conclusions

We used biomarkers from DTI and CSF to classify patients into more homogeneous groups. Other biomarkers become more clearly separated using this paradigm. The presence of dual pathologies could be a reason for the failure of a number of studies in AD using monotherapy to reduce amyloid. Having the ability to identify the patients with AD and SIVD during life, and the ability to identify them at an earlier stage in the disease, could lead to more rational treatment trials. Machine learning algorithms such as Random Forest, can further improve the probability of reaching the correct diagnosis, forming a pathway to precision medicine in dementia. This study was done in a single center cohort with a small sample size. Before it can be implemented on a larger scale and used in treatment trials, it will need to be replicated in a larger number of patients from multiple centers.

## Figures and Tables

**Table 1:** Biomarker Features used to make Diagnoses.

	SIVD *	AD	MX	LA
Clinical				
Hyperreflexia	+	−	+	−
Imbalance	+	−	+	−
Neuropsych				
Executive	+	−	−	−
Memory	−	+	+	−
MRI				
WMH (FLAIR)	+	−	+	+
MD/RD (DTI)	+	−	+	−
CSF				
Amyloid 42/40	−	+	+	−
Phospho-Tau	−	+	+	−
Albumin Index	+	−	+	−

See text for definitions of diagnostic categories.

**Table 2:** Diagnosis Count based on Clinical and Biological Criteria.

Diagnostic Criteria	SIVD/BD	AD	MX	MI	LA	Total
Clinical	59	33	9	17	29	147
Biological	53	25	22	18	29	147

**Table 3:** Major features in the different patient groups separated by the biological biomarkers.

	Biological Diagnosis				
	LA	MX	SVD	AD	Control
Age at Baseline	62.79 ± 10.21 *	73.09 ± 6.53	65.64 ± 13.29	68.76 ± 8.15	58.68 ± 15.83 **
T-memory	47.31 ± 9.42	34.24 ± 9.42	42.80 ± 12.36	29.64 ± 7.96	53.56 ± 10.47
T-executive	46.93 ± 7.93	42.60 ± 6.34	40.62 ± 6.68	43.40 ± 8.61	50.28 ± 6.01
PSMD Z-Score	1.02 ± 1.47	3.20 ± 1.65	2.94 ± 2.12	0.74 ± 0.56	−0.03 ± 0.93
CSF MMP-1	26.18 ± 17.36	20.20 ± 13.67	26.49 ± 23.66	22.75 ± 0.67	14.47 ± 10.24
CSF MMP-10	68.70 ± 40.94	104.40 ± 37.04	71.70 ± 36.07	103.94 ± 72.59	52.98 ± 23.71
Ptau	46.05 ± 16.16	118.86 ± 57.82	47.43 ± 19.56	87.75 ± 37.51	55.80 ± 22.57

* All numbers are mean ± standard deviation. ** Statistical significance was present at the *p* < 0.05 level for all variables.

## References

[1] Hachinski V., Iadecola C., Petersen R.C., Breteler M.M., Nyenhuis D.L., Black S.E., Powers W.J., DeCarli C., Merino J.G., Kalaria R.N. (2006). National Institute of Neurological Disorders and Stroke-Canadian Stroke Network vascular cognitive impairment harmonization standards. Stroke.

[2] Snyder H.M., Corriveau R.A., Craft S., Faber J.E., Greenberg S.M., Knopman D., Lamb B.T., Montine T.J., Nedergaard M., Schaffer C.B. (2015). Vascular contributions to cognitive impairment and dementia including Alzheimer’s disease. Alzheimers Dement..

[3] Rosenberg G.A., Wallin A., Wardlaw J.M., Markus H.S., Montaner J., Wolfson L., Iadecola C., Zlokovic B.V., Joutel A., Dichgans M. (2016). Consensus statement for diagnosis of subcortical small vessel disease. J. Cereb. Blood Flow Metab..

[4] Jack C.R., Bennett D.A., Blennow K., Carrillo M.C., Dunn B., Haeberlein S.B., Holtzman D.M., Jagust W., Jessen F., Karlawish J. (2018). NIA-AA Research Framework: Toward a biological definition of Alzheimer’s disease. Alzheimers Dement..

[5] Kövari E., Gold G., Giannakopoulos P., Herrmann F.R., Bouras C. (2007). Identification of Alzheimer and vascular lesion thresholds for mixed dementia. Brain.

[6] Schneider J.A., Arvanitakis Z., Bang W., Bennett D.A. (2007). Mixed brain pathologies account for most dementia cases in community-dwelling older persons. Neurology.

[7] Sonnen J.A., Santa Cruz K., Hemmy L.S., Woltjer R., Leverenz J.B., Montine K.S., Jack C.R., Kaye J., Lim K., Larson E.B. (2011). Ecology of the aging human brain. Arch Neurol..

[8] Toledo J.B., Arnold S.E., Raible K., Brettschneider J., Xie S.X., Grossman M., Monsell S.E., Kukull W.A., Trojanowski J.Q. (2013). Contribution of cerebrovascular disease in autopsy confirmed neurodegenerative disease cases in the National Alzheimer’s Coordinating Centre. Brain.

[9] Snowdon D.A., Greiner L.H., Riley K.P., Markesbery W.R., Mortimer J.A. (1997). Brain infarction and the clinical expression of Alzheimer disease: The Nun Study. JAMA.

[10] Rosenberg G.A., Prestopnik J., Adair J.C., Huisa B.N., Knoefel J., Caprihan A., Gasparovic C., Thompson J., Erhardt E.B., Schrader R. (2015). Validation of biomarkers in subcortical ischaemic vascular disease of the Binswanger type: Approach to targeted treatment trials. J. Neurol. Neurosurg. Psychiatry.

[11] Erhardt E.B., Pesko J.C., Prestopnik J., Thompson J., Caprihan A., A Rosenberg G. (2018). Biomarkers identify the Binswanger type of vascular cognitive impairment. J. Cereb. Blood Flow Metab..

[12] Chui H.C., Ramirez-Gomez L. (2015). Clinical and imaging features of mixed Alzheimer and vascular pathologies. Alzheimers Res. Ther..

[13] Duering M., Finsterwalder S., Baykara E., Tuladhar A.M., Gesierich B., Konieczny M.J., Malik R., Franzmeier N., Ewers M., Jouvent E. (2018). Free water determines diffusion alterations and clinical status in cerebral small vessel disease. Alzheimers Dement..

[14] Jack C.R., Holtzman D.M. (2013). Biomarker modeling of Alzheimer’s disease. Neuron.

[15] Maillard P., Seshadri S., Beiser A., Himali J.J., Au R., Fletcher E., Carmichael O., Wolf P.A., DeCarli C. (2012). Effects of systolic blood pressure on white-matter integrity in young adults in the Framingham Heart Study: A cross-sectional study. Lancet Neurol..

[16] Rosenberg G.A., Kornfeld M., Stovring J., Bicknell J.M. (1979). Subcortical arteriosclerotic encephalopathy (Binswanger): Computerized tomography. Neurology.

[17] Caplan L.R. (1995). Binswanger’s disease-revisited. Neurology.

[18] Huisa B.N., Rosenberg G.A. (2014). Binswanger’s disease: Toward a diagnosis agreement and therapeutic approach. Expert Rev. Neurother..

[19] McKhann G., Drachman D., Folstein M., Katzman R., Price D., Stadlan E.M. (1984). Clinical diagnosis of Alzheimer’s disease: Report of the NINCDS-ADRDA Work Group under the auspices of Department of Health and Human Services Task Force on Alzheimer’s Disease. Neurology.

[20] McKhann G.M., Knopman D.S., Chertkow H., Hyman B.T., Jack C.R., Kawas C.H., Klunk W.E., Koroshetz W.J., Manly J.J., Mayeux R. (2011). The diagnosis of dementia due to Alzheimer’s disease: Recommendations from the National Institute on Aging-Alzheimer’s Association workgroups on diagnostic guidelines for Alzheimer’s disease. Alzheimers Dement..

[21] Hachinski V.C., Potter P., Merskey H. (1987). Leuko-araiosis. Arch. Neurol..

[22] Iadecola C., Anrather J. (2011). The immunology of stroke: From mechanisms to translation. Nat. Med..

[23] Rosenberg G.A. (2009). Matrix metalloproteinases and their multiple roles in neurodegenerative diseases. Lancet Neurol..

[24] Rosenberg G.A. (2016). Matrix Metalloproteinase-Mediated Neuroinflammation in Vascular Cognitive Impairment of the Binswanger Type. Cell. Mol. Neurobiol..

[25] Raja R., Rosenberg G.A., Caprihan A. (2017). MRI measurements of Blood-Brain Barrier function in dementia: A review of recent studies. Neuropharmacology.

[26] Bjerke M., Zetterberg H., Edman A., Blennow K., Wallin A., Andreasson U. (2011). Cerebrospinal fluid matrix metalloproteinases and tissue inhibitor of metalloproteinases in combination with subcortical and cortical biomarkers in vascular dementia and Alzheimer’s disease. J. Alzheimers Dis..

